# *Arthrobacter nitrophenolicus* sp. nov*.* a new 2-chloro-4-nitrophenol degrading bacterium isolated from contaminated soil

**DOI:** 10.1007/s13205-012-0066-4

**Published:** 2012-05-05

**Authors:** Pankaj Kumar Arora, Rakesh Kumar Jain

**Affiliations:** 1 Environmental Biotechnology, Microbial Type Culture Collection and Gene Bank (MTCC), Institute of Microbial Technology (CSIR), Sector-39A, Chandigarh, India; 2 Department of Plant Sciences, School of Life Sciences, University of Hyderabad, P.O. Central University, Hyderabad, 500 046 India

**Keywords:** Biodegradation, 2-Chloro-4-nitrophenol, 4-Nitrophenol, 3-Methyl-4-nitrophenol

## Abstract

Strain SJCon^T^, a 2-chloro-4-nitrophenol (2C4NP) degrading bacterium, was isolated from soil collected from a pesticide-contaminated site in Punjab, India. The strain, which stained Gram positive, displayed a rod-coccus life cycle, and possessed a type A3_α_ peptidoglycan (l-Lys–l-Ala_3_), MK-9(H2) as the major menaquinone, anteiso-C15 and iso-C15:0 as the major cellular fatty acids, and diphosphatidylglycerol, phosphatidylglycerol, phosphatidylinositol and a glycolipid as the major polar lipids, showed morphological and chemotaxonomic properties consistent with those reported for members of the genus *Arthrobacter.* Phylogenetic analysis of the 16S rRNA gene sequence of strain SJCon^T^ confirmed that it was a member of this genus with *Arthrobacter globiformis* DSM 20124^T^ being the closest relative (sequence similarity of 97 %). The DNA G + C content of strain SJCon^T^ was 69 ± 1 mol% and DNA homology with *A. globiformis* DSM 20124^T^ was 45 %, suggesting that strain SJCon^T^ represented a novel species of the genus *Arthrobacter*, which we have named *Arthrobacter nitrophenolicus* sp. nov The type strain is SJCon^T^ (=MTCC 10104^T^ =DSM 23165^T^).

Genus *Arthrobacter* was first proposed by Conn and Dimmick (1947) with the description of the type species *Arthrobacter globiformis.* Subsequently, Koch et al. (1995) emended the description with the reclassification of *Micrococcusagilis* as *Arthrobacteragilis*. The currently validated 78 members of genus *Arthrobacter* are members of phylum *Actinobacteria*, order *Actinomycetales*, family *Micrococcaceae*, and are characterized by the presence of a rod–coccus growth cycle and genomes with high G + C content (59–66 mol%) (Keddie et al. 1986)*.* Members of genus *Arthrobacter* stain Gram positive are catalase positive and are sub-divided on the basis of the lysine-containing peptidoglycan into two groups, A3α and A4α (Schleifer and Kandler 1972; Stackebrandt et al. 1983; Keddie et al. 1986; Koch et al. 1995).

The primary habitat of *Arthrobacter* is soil and interestingly *Arthrobacter* strains with the ability to degrade nitrophenols and/or chlorophenols which include *Arthrobacter chlorophonolicus* A6 (Westerberg et al. 2000), *Arthrobacter ureafaciens*, strain CPR706 (Bae et al. 1996), *Arthrobacter citrus* (Karigar et al. 2006), *Arthrobacter protophormae* strain RKJ100 (Chauhan et al. 2000), *Arthrobacter* sp. strain JS443 (Jain et al. 1994) and *Arthrobacter aurescens* TW17 (Hanne et al. 1993) have all been isolated from pesticide-contaminated soil. We had previously reported for the first time on the isolation of *Arthrobacter* strain SJCon that degraded 2-chloro-4-nitrophenol (2C4NP) (Arora and Jain 2011). Chlorohydroquinone was identified as a major intermediate product which was further degraded via formation of maleylacetate (Arora and Jain 2011). As strain SJCon^T^ is a potential degrader of various nitrophenolic compounds including 2-chloro-4-nitrophenol, 4-nitrophenol and 3-methyl-4-nitrophenol, and it has the potential for use in the bioremediation of nitrophenolic contaminated sites. In this communication, we report on the chemotaxonomic and genotypic properties of the strain and designate it as a new species of genus, *A. nitrophenolicus* sp nov. (The type strain is SJCon^T^ =MTCC 10104^T^ =DSM 23165^T^).

The method for isolating strain SJCon^T^ from a pesticide-contaminated soil by an enrichment method using 2-chloro-4-nitrophenol as sole carbon source (Sigma-Aldrich, GmbH, Steinheim, Germany) has been reported previously (Arora and Jain 2011).

Colony morphology was examined on Nutrient agar plates after incubation at 30 °C for 24 h. Cell morphology was examined by light microscopy (Zeiss) at ×1000 and motility was checked using the method described by Skerman (1967). Gram staining was performed using HiMedia Gram Staining kit (HiMedia, India) according to the manufacturer’s instructions. Strain SJCon^T^ had morphological characteristics consistent with members of the genus *Arthrobacter.* Cells exhibited a rod–coccus growth cycle, were non-motile, did not form spores and stained Gram positive.

Growth at different temperatures (between 4 and 50 °C) was determined using Nutrient agar plates. Strain SJCon^T^ was streaked on nutrient agar plates, incubated at different temperatures and the growth on the plate scored. The growth in nutrient broth containing different NaCl concentrations (0.5–7 %) was monitored by measuring optical density at 600 nm in a Lambda 35 spectrophotometer (Perkin Elmer). Growth at different pH (pH 4.5–12) was tested in nutrient broth after the pH had been adjusted using appropriate buffers as described previously (Arora et al. 2011). Growth occurred between 10 and 40 °C with the optimum temperature for growth at 30 °C. The optimum pH for growth was 7 and no growth occurred below pH 6 or above pH 10. NaCl was not required for growth, but was tolerated up to 4 %.

Hydrolysis of gelatin, casein, starch, Voges-Proskauer, methyl red, oxidation-fermentation tests, catalase and oxidase activities, growth on Simmon’s citrate and MacConkey agar, production of H_2_S and indole, reduction of nitrate and acid production from carbohydrates were determined as described previously (Arora et al. 2011).

A summary of the results from the phenotypic tests is presented in Table 1 and listed in the species description.

**Table 1:** Differentiating characteristics of strain *Arthrobacter nitrophenolicus* strain SJCon^T^ and *Arthrobacter globiformis* DSM 20124^T^

Characteristics features	*Arthrobacter nitrophenolicus*	*Arthrobacter globiformis*
Type strains	SJCon^T^ =MTCC 10104^T^ =DSM 23165^T^	DSM 20124^T^
Sample source	Pesticide-contaminated soil	Soil
16S rRNA gene accession number	GQ927310	M23411
Mol% G + C	69 ± 1 mol%	62.0 %
DNA homology (%) of strain SJCon^T^ with:	100	45
NaCl range	0–4 %	0–5 %
Starch hydrolysis	–	+
Casein hydrolysis	+	–
Oxidation of substrates		
d-Mannitol	+	–
Utilization of various nitro aromatics as sole carbon and energy source		
2-Chloro-4-nitrophenol	+	–
4-Nitrophenol	+	–
3-Methyl-4-nitrophenol	+	–

Both species produce white colonies, have a rod-coccus life cycle, grow between pH 6 and 10, do not reduce nitrate, hydrolyse gelatin and utilise l-arabinose and glucose

The utilization of carbon and energy sources was tested using Biolog GP2 Microplates (Hayward, CA). For this, the inoculum was prepared by re-suspending Nutrient agar grown colonies to a turbidity equivalent to 0·5 McFarland units and the GP2 Microplates inoculated following the manufacturer’s instructions. The plates were incubated at 30 °C for 24 h and the results read using a MicroPlate Reader equipped with Microlog 4.2 software. The results are listed in the species description.

Menaquinones, fatty acids, polar lipids and peptidoglycan were analyzed by standard methods (Arora et al. 2011). The major menaquinone was identified as MK-9(H_2_), a characteristic chemotaxonomic marker of the genus *Arthrobacter*. The fatty acid profile comprises C_16:0_ (6.48 %), C_18:0_ (1.61 %), iso-C_14:0_ (0.93 %), iso-C_15:0_ (16.33 %), anteiso-C_15:0_ (44.18 %), iso-C_16:0_ (9.25 %), iso-C_17:0_ (5.61 %) and anteiso-C_17:0_ (15.19 %). The major polar lipids were diphosphatidylglycerol (DPG), phosphatidylglycerol (PG), phosphatidylinositol (PI) and a glycolipid. The peptidoglycan was identified as type A3_*α*_ (l-Lys–l-Ala_3_).

DNA extraction and purification have been described previously (Arora and Jain 2011). The 16S rRNA gene was amplified using universal bacterial primers 8F (5′-AGA GTT TGA TCC TGG CTC AG-3′) and 1492R (5′-GGT TAC CTT GTT ACG ACT T-3′) (Arora et al. 2011) and sequenced using n ABI automated sequencer (Applied Biosystems, USA). The sequence (1,446 bp with GenBank accession number GQ927310) was compared against sequences available in the GenBank database (version 188.0) using EzTaxon server 2.1, the sequences of the nearest phylogentic members downloaded, aligned with those of related *Arthrobacter* species and a phylogenetic tree constructed using the neighbour-joining method as implemented in MEGA (Tamura et al. 2007) and Bioedit (Hall 1999). During phylogenetic reconstruction, all ambiguous nucleotides were excluded from the analysis and a total of 1,270 nucleotides were used in the final analysis. Phylogenetic analysis showed that strain SJCon^T^ was a member of the genus *Arthrobacter* and showed the highest similarity to *A. globiformis* DSM 20124^T^ and related members of the ‘globiformis’ group (average sequence similarity of 97%) (Fig. 1).

**Fig. 1:**
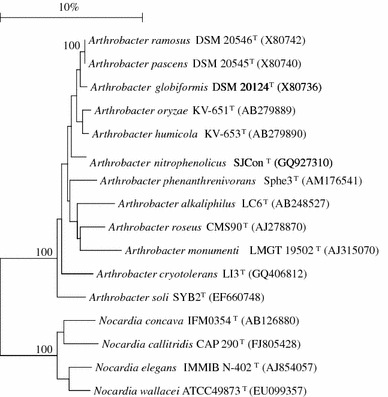
The phylogenetic position of *Arthrobacter nitrophenolicus* strain SJCon^T^ within the radiation of members of genus *Arthrobacter* based on 16S rRNA gene. The type strains are indicated with the *letter*^*T*^ and the accession numbers are shown in *brackets*. Bootstrap values >95 % (expressed as percentages of 1000 replications) are indicated at the *branch points*

The G + C mol% content of the genomic DNA was determined in a Lambda 35 spectrophotometer (Perkin Elmer, Waltham, MA, USA) using the thermal denaturation (Tm) method and determined to be 69 ± 1 mol%.

DNA hybridization was performed using Biotin DecaLabelTM Kit and Biotin Chromogenic Detection Kit (Fermentas Life Sciences) following the manufacturer’s instruction. The DNA–DNA relatedness value of strain SJCon^T^ with *A. globiformis* DSM 20124^T^ was found to be 45 %.

Strain SJCon^T^ degrades 2-chloro-4-nitrophenol, 4-nitrophenol and 3-methyl-4-nitrophenol. It possesses morphological characteristic of rod-coccus life cycle, which is consistent with that reported for members of the genus *Arthrobacter.* It’s affiliation to this genus is supported by the chemotaxonomic traits and 16S rRNA phylogeny. In addition, the presence of cell-wall peptidoglycan type A3_*α*_ and menaquinone MK-9(H_2_) supports the placement of strain SJCon^T^ within the “globiformis” group. However, the large phylogenetic distance, the low DNA homology and the higher mol % G + C content and a number of phenotypic traits (Table 1) differentiate *s*train SJCon^T^ from *A. globiformis* DSM 20124^T^ and members of the “globiformis” group. The fatty acid composition of strain SJcon^T^ was differed from the fatty acid composition of *A*. *globiformis* DSM 20124^T^ (Table 2). The fatty acid profile of strain SJCon^T^ was comprises C_16:0_ (6.48 %), C_18:0_ (1.61 %), iso-C_14:0_ (0.93 %), iso-C_15:0_ (16.33 %), anteiso-C_15:0_ (44.18 %), iso-C_16:0_ (9.25 %), iso-C_17:0_ (5.61 %) and anteiso-C_17:0_ (15.19 %). On the basis of these polyphasic differences, strain SJCon^T^ represents a novel species of the genus *Arthrobacter*, for which the name *Arthrobacter nitrophenolicus* sp. nov. is proposed. The type strain is SJCon^T^ (=MTCC 10104^T^ =DSM 23165^T^).

**Table 2:** Fatty acid profile of *Arthrobacter nitrophenolicus* strain SJCon^T^ and *Arthrobacter globiformis* DSM 20124^T^ (Data from present study)

Fatty acid	*Arthrobacter nitrophenolicus*	*A. globiformis*
14:0 iso	0.93	ND
15:0 iso	16.33	13.3
15:0 anteiso	44.18	55.2
16:0 iso	9.25	21.0
16:0	6.48	ND
17:0 iso	5.61	ND
17:0 anteiso	15.19	10.50
18:0	1.61	ND

*ND* Not detected

## Description of *Arthrobacter nitrophenolicus* sp. nov.

(Nitr.o.phen.o.li.cus.M.L. adj. *Nitro* containing nitrogen, N.L. n. *phenol* phenol; M.L. adj. *nitrirophenolicus* relating to nitrophenol).

Cells are Gram-positive, catalase-positive, oxidase-negative, non-spore-forming, non-motile and exhibit a rod–coccus growth cycle. The optimal temperature for growth is 30 °C (temperature growth range of 10–40 °C) and pH 7.0 (pH growth range of pH 6–10). The cells grow on nutrient broth medium with 4 % NaCl, and degrade 2-chloro-4-nitrophenol, 4-nitrophenol and 3-methyl-4-nitrophenol. Chlorohydroquinone, a major intermediate product of degradation of 2-chloro-4-nitrophenol, which is further degraded via formation of maleylacetate. It hydrolyses gelatin and casein but not starch. No growth occurs on Simmon Citrate and Mac Conkey Agar. Chlorohydroquinone is negative for indole production, H_2_S production, nitrate reduction, Voges-Proskaur test, methyl red, and oxidation-fermentation tests; It produce acids from l-arabinose, d-glucose, and d-manitol, and oxidizes the following substrates in Biolog GN2 Microplates: arbutin, d-cellobiose, d-fructose, d-galactose, d-gluconic acid, α-d-glucose, m-inositol, α-d-lactose, maltotriose, d-mannitol, d-mannose, d-melizitose, d-ribose, salicin, d-sorbitol, sucrose, turanose, d-xylose, acetic acid, β-hydroxybutyric acid, γ-hydroxybutyric acid, α-ketoglutaric acid, l-lactic acid, l-malic acid, pyruvatic acid methyl ester, pyruvic acid, l-alaninamide, d-alanine, l-alanine, L-alanyl-Glycine, l-asparagine, l-glutamic acid, glycyl-l-glutamic acid, L-pyroglutamic acid, l-serine, putrescine, glycerol, adenosine, inosine. C_16:0_ (6.48), C_18:0_ (1.61 %), iso-C_14:0_ (0.93 %), iso-C_15:0_ (16.33 %), anteiso-C_15:0_ (44.18 %), iso-C_16:0_ (9.25 %), iso-C_17:0_ (5.61 %) and anteiso-C_17:0_ (15.19 %) are the major fatty acids. The major polar lipids are diphosphatidylglycerol, phosphatidylglycerol, phosphatidylinositol and a glycolipid. The major menaquinone detected is MK-9(H_2_) and the peptidoglycan-type is A3_α_. The DNA G + C content of strain SJCon^T^ is 69 ± 1 mol%.

The type strain SJCon^T^ =MTCC 10104^T^ =DSM 23165^T^ was isolated from a pesticide-contaminated soil sample collected in Punjab State, India.
